# Tuberculosis caused by *Mycobacterium caprae* in a camel (*Camelus dromedarius*)

**DOI:** 10.1186/s12917-020-02665-0

**Published:** 2020-11-10

**Authors:** J. A. Infantes-Lorenzo, B. Romero, A. Rodríguez-Bertos, A. Roy, J. Ortega, L. de Juan, I. Moreno, M. Domínguez, L. Domínguez, J. Bezos

**Affiliations:** 1 Servicio de Inmunología Microbiana, Centro Nacional de Microbiología, Instituto de Salud Carlos III, Majadahonda, Madrid, Spain; 2 VISAVET Health Surveillance Centre, Complutense University of Madrid, Madrid, Spain; 3 Department of Animal Medicine and Surgery, Faculty of Veterinary Medicine, Complutense University of Madrid, 28040 Madrid, Spain; 4 BIOFABRI S.L., Porriño, Pontevedra, Spain; 5 Department of Animal Health, Faculty of Veterinary Medicine, Complutense University of Madrid, 28040 Madrid, Spain

**Keywords:** Animal tuberculosis, Diagnostic tests, *Mycobacterium caprae*, Old World Camelids, Pathology

## Abstract

**Background:**

Animal tuberculosis (TB) is distributed worldwide and has a wide range of wild and domestic reservoirs. Few studies concerning TB in camelids have been published in the last decade, particularly as regards Old World Camelids (OWC), but the increase in reports of TB outbreaks in these species in recent years suggests a high susceptibility to the infection.

**Case presentation:**

We studied a dromedary camel (*Camelus dromedarius*) herd (*n* = 24) in which a *Mycobacterium caprae* infection was detected. The TB infection was confirmed in one animal at necropsy through the detection of TB lesions, mainly in the abdominal organs, and the subsequent isolation of *M. caprae* (SB0157 spoligotype). The whole herd was additionally tested using cellular and humoral based diagnostic techniques. The intradermal tuberculin test results were compared with those obtained using P22 ELISA for the detection of specific antibodies against the *M. tuberculosis* complex. The TB infected animal was a positive reactor to both the intradermal tuberculin tests and P22 ELISA, while the others were negative to all the diagnostic tests.

**Conclusion:**

The present study found *M. caprae* infection in OWC. This is the first report of *M. caprae* infection in an OWC not living in a zoo. Since the animal was born in the herd and fed with goat’s milk, this practice was suspected to be the potential source of TB infection, which was not confirmed in the other animals present in the herd. Moreover, our results highlight that the intradermal tuberculin test and the P22 ELISA could be valuable tools for the diagnosis of TB in OWC.

**Supplementary Information:**

The online version contains supplementary material available at 10.1186/s12917-020-02665-0.

## Background

Tuberculosis (TB) in animals is a zoonotic disease caused by members of the *Mycobacterium tuberculosis* complex (MTBC), mainly *M. bovis* and *M. caprae*, and continues to be one of the most widespread animal diseases in the world [1]. Mycobacteria belonging to the MTBC have been isolated from a great variety of domestic and wild animals, including both South American Camelids (SAC) (llama, alpaca and vicuna) and Old World Camelids (OWC) (Bactrian camel and dromedary) [2–6]. Another member of the MTBC, *M. microti*, has been described as a causative agent of camel TB [6]. Moreover, *M. kansasii, M. aquae, M. fortuitum, M. smegmatis* and *M. avium* complex have also been isolated from camelids’ TB [7].

While TB infection in SAC has been the object of study in several published manuscripts, the information concerning TB in OWC is more limited. Most of the outbreaks previously reported were caused by *M. bovis,* potentially owing to the fact that the OWC had been imported from endemic TB regions [8, 9] or had come into contact with infected cattle [9, 10]. With regard to the *M. caprae* infection, to the best of the authors’ knowledge, only one case has been previously reported in a dromedary living in a zoo in Slovenia [11]. There are many potential sources of infection for OWC, and transmission may occur through contact with infected livestock or wild animals [8]. Furthermore, these animals may come into regular contact with humans and other susceptible species, since they are used for touristic camel rides in certain countries and they are present in zoos and farm-schools. Indeed, a recent study in Iran identified camels as one of the most important sources of infections and diseases for humans [12].

The diagnosis of TB in OWC is based on various ante-mortem (intradermal tuberculin test, serology) and post-mortem (pathology and bacteriological culture) techniques. However, the accurate ante-mortem diagnosis of TB is generally difficult, and few studies reporting the performance of TB diagnostic tests using different interpretation criteria in live OWC are available [4, 13]. The use of the comparative intradermal tuberculin (CIT) test rather than a single intradermal tuberculin (SIT) test is usually preferred as a first option for diagnosis owing to the potential interference caused by environmental mycobacteria or *Corynebacterium pseudotuberculosis* infections [8, 14]. In addition, some studies have highlighted the potential diagnostic value of the serological tests, since they have proved to have a higher sensitivity in the case of OWC than tests based on cell-mediated immunity [4, 15]. The necropsy of infected camels has shown different patterns of TB lesions, most of them in the respiratory tract [9], although the presence of lesions in the digestive tract and other organs has been also reported, suggesting the importance of the oral route for transmission in camels [7, 10].

In the present study, we report the first case of TB caused by *M. caprae* in a camel (*C. dromedarius*) in Spain. In addition, since there is little data on TB diagnostic tests in OWC, the diagnostic techniques reported herein were performed on and evaluated for the whole herd.

## Case presentation

### Herd situation and diagnostic tests

The study was performed in a camelid herd used for touristic rides located in the South-East of Spain. The herd consisted of 24 animals, 23 *Camelus dromedarius* and one cross-breed camel. In December 2016, the herd was subjected to annual routine testing by means of a SIT test, which resulted in one positive animal (Animal 1). The whole herd was then studied for the following 30 months (starting in December 2016 and finishing in April 2019) using various TB diagnostic tests that were performed as briefly detailed below. The animals included in this study were not experimental animals. All handling and procedures were carried out in accordance with Spanish legislation.

#### Intradermal tuberculin tests

The SIT and CIT tests were carried out in the cervical region using the commercial bovine and avian PPDs (bPPD and aPPD, respectively) (CZ Vaccines S.A., Porriño, Spain) and were interpreted as described elsewhere [16, 17]. Briefly, the animals were inoculated with 0.1 ml of bPPD on the left-hand side of the thorax area, just behind of scapula (SIT test), and with 0.1 ml of a PPD on the right-hand side (CIT test) using a McLintock syringe (Bar Knight McLintock Limited, Clydebank, UK). The results of the tests were determined by measuring the increase in the skin-fold thickness 72 h later and observing the local clinical signs.

#### Serological tests

An indirect ELISA that detects antibodies against P22, a protein complex obtained by affinity chromatography from bPPD, was used [18]. The ELISA was performed as described previously [19], with minor modifications: detection antibody (Goat anti-Llama IgG (H + L)-HRP) was used at 1:2000 and the substrate was incubated for 15 min in darkness and under room temperature conditions. The sample results were expressed as an ELISA percentage (E %), calculated by employing the following formula: [sample E% = (mean sample OD/ 2 x mean of negative control OD) × 100]. Serum samples with E% values of between 100 and 150 were considered inconclusive, while values greater than 150 were considered positive.

The Dual Path Platform (DPP) assay (Chembio Diagnostic Systems, Inc., USA) was also used, following manufacturer’s instructions.

#### Post-mortem examination and bacteriological culture

Before euthanasia, animals were sedate by intravenous injection of xylazine (0.25 mg/kg body weight). Once they were in deep sedation, euthanasia was conducted by an intravenous injection dose of pentobarbital sodium (Dolethal, Vetoquinol, Spain, 100 mg/kg body weight). In order to minimize the stress, animals were previously moved to a quiet and peaceful environment and further away from the rest of the herd.

Tissue samples collected from animals subjected to post-mortem analysis were fixed in 10% neutral buffered formalin for 24 h at room temperature. The samples were then embedded in paraffin wax using an automatic tissue processor. Four micron thick sections were obtained and haematoxylin–eosin and Ziehl–Neelsen stained. In addition, fresh tissue samples from lung and lymph nodes from the head and thorax were cultured using BACTEC MGIT 960 Mycobacterial Detection System (Becton Dickinson, USA). A urine sample was also collected from one SIT test reactor animal and was used for bacteriological culture as described previously. Isolates were identified and characterised by means of Direct Variable Repeat (DVR)-spoligotyping [20]. The samples were cultured in parallel on Columbia Agar media plates with 5% of sheep’s blood (BioMèrieux, Madrid, Spain) for the isolation of *Corynebacterium pseudotuberculosis*.

### Results by testing events

#### December 2016

In the first testing event, all the animals were subjected to SIT tests and only one (Animal 1) was a positive reactor (Table 1), with an increase in skin fold thickness of 6 mm at the bPPD injection site. On that occasion, only this animal was tested using serology, and was also positive to the P22 ELISA but negative to the DPP assay. Based on these results, Animal 1 was suspected to be TB-infected and was kept in isolation in a different facility.

**Table 1:** Positive animals out of the total number of camels on the farm (*n* = 24) to each diagnostic test in the different testing events throughout the study

Test	December 2016^**e**^	February 2017	October 2017	February 2018	April 2018	October 2018	March 2019
**SIT**^**a**^	1/24 (Animal 1)	1/1 (Animal 1)	ND	ND	2/23(Animals 2 and 4)	ND	0/20
**CIT**^**b**^	ND^f^	1/1 (Animal 1)	ND	ND	0/23	ND	0/20
**Serology P22**^**c**^	1/1 (Animal 1)	1/24* (Animal 1)	1/24* (Animal 1)	0/23*	0/23*	ND	0/20*
**DPP**^**d**^	0/1	0/24	0/24	0/23	0/23	ND	0/20
**Euthanasia/natural death**	–	–	–	1 animal (euthanasia, Animal 1)	2 animals (euthanasia, Animals 4 and 5)	1 animal natural death (Animal 6)	–
**Pathology**	ND	ND	ND	1/1 (Animal 1 TB-compatible lesions)	0/2	0/1	ND
**Bacteriology**	ND	ND	ND	1/1 (Animal 1-*M. caprae*)	0/2	0/1	ND

* Animals 2 and 3 attained inconclusive results

^a^SIT: Single intradermal test. Positive if a bovine reaction of 2 or more mm or the presence of clinical signs such as oedema, exudation, necrosis, pain or inflammation at the injection site were observed

^b^CIT: comparative intradermal test. Positive if a bovine reaction of 2 or more mm was observed that was greater than the avian reaction, or if the presence of clinical signs at the injection site of the bovine PPD were observed

^c^ P22 ELISA: positive if the E% is greater than 150% and inconclusive if the E% is between 100 and 150%

^d^DPP: Dual Path Platform

^e^Serology only performed on the SIT positive animal (Animal 1)

^f^ ND: not determined

Moreover, a complete blood count and biochemical analysis were carried out on Animal 1. This animal had an increased number of total leukocytes (14,400/μL), which particularly affected the lymphocytes (4896/μL) when compared to the control (6150/μL and 1783/ μL, respectively). The Urea and ASGT-GOT were also higher (Supplementary material 1) than in the control.

#### February 2017

The whole herd (*n* = 24) was tested using the P22 ELISA and the DPP assay. Animal 1 was again positive to the P22 ELISA, while two animals (Animals 2 and 3) were considered inconclusive reactors since their OD values were very close to the cut-off point. All the animals were negative to the DPP assay. Animal 1 was tested again using the SIT and CIT tests, and was found to have an increase in skin fold thickness of 1 mm and 5 mm at the aPPD and bPPD injection sites, respectively (Table 1). A urine sample was also collected from this animal for bacteriological culture, although no mycobacteria were subsequently isolated.

#### October 2017

In this herd testing event, the whole herd was tested using both P22 ELISA and the DPP asssay. Animal 1 was positive to the P22 ELISA, whilst the remaining camels did not react. All the animals were negative to the DPP assay.

#### February 2018

**Fig. 1:**
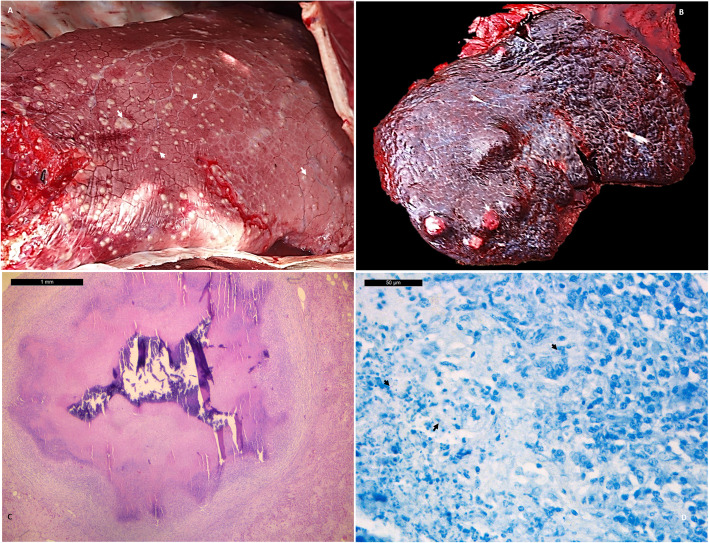
Camel: Gross lesions: **a** Lung: Multifocal to coalescent pulmonary granulomas (arrows) with haphazar distribution on parenchyma lobes; **b** Spleen: Intraparenchymatous granulomas of various size (from 0,5 to 2 cm of diameter - arrows); Histological lesions: **c** Histological apperance of granuloma charecterized by central necrosis with dystrophic mineralization (*) compatible with granuloma type III (Wangoo et al., 2005). Bar = 1 mm; **d** Small number of pink bacilli (arrows) in the necrotic area (1 bacillus / HPF / 10 fields) can be observed (little bacillary lesion). Zielh-Neelsen technique. Bar = 50 μm

Animal 1 was euthanized owing to its poor body condition (two weeks before euthanasia, the animal lost weight, had dull hair and was apathetic), and was subjected to a post-mortem analysis. At necropsy, a slight amount of yellowish abdominal fluid was observed in the abdominal (ascites), thoracic (hydrothorax) and pericardial (hydropericardias) cavities, and numerous nodules of varying sizes (0.5–3 cm of diameter) were found inside the spleen (Fig. 1b) and liver. These formations were characterised by well-delimitated creamy nodes, most of them close to the surface. The pulmonary parenchyma had multifocal miliar nodes with an irregular distribution throughout the lobes (Fig. 1a), and covered some areas of serosa membranes of peritoneum and parietal pleura. The miliary nodes were cut into 2–3 mm thick slices and macroscopic inspection revealed calcified caseum material with a multifocal to coalescent whitish, hard and rough appearance. A generalised hypertrophy of the lymph nodes was observed, mainly in the mesenteric and mediastinal lymph nodes. According to the distribution of gross lesions, the animal had a primary TB infection with a chronic and generalised phase, which also affected the serosal membrane. A histological examination revealed granulomatous lesions in the spleen and liver and less advanced lesions in the pulmonary parenchyma. With regard to the classification described by Wangoo and collaborators for bovine TB [21], the lesions were categorized as Type IV granulomatous, characterised by multiple coalescent calcified lesions with caseous central necrosis and with a very small number of Langerhans cells (Fig. 1c). The Ziehl-Neelsen technique showed a small number of positive bacteria (low bacillary load– less than 50 acid-fast bacilli) (Fig. 1d). Animal 1 showed gross TB compatible lesions, and the histology confirmed the presence of TB granulomas in different locations, with more evolved nodes in the abdominal organs, liver and spleen in comparison with those in the pulmonary parenchyma.

In addition, tissue samples from the lungs and lymph nodes from Animal 1’s head and thorax were used for bacteriological culture, and a *M. caprae* strain (SB0157 spoligotype) was subsequently isolated and identified.

#### April 2018

As a consequence of the confirmation of the *M. caprae* infection in Animal 1, the whole herd was again subjected to the SIT/CIT tests. Two additional animals were found to be positive when using the severe interpretation of the SIT test (> 2 mm), but were negative to the CIT test (Animals 2 and 4). All the animals were negative to both the P22 ELISA and DPP serological tests.

The SIT test results (severe interpretation), and its poor body condition led Animal 4 to be euthanized and subjected to post-mortem analysis. This animal did not have TB-compatible lesions, although it was found to have several other damages, including a severe micronodular cirrhosis and chronic interstitial pneumonia, which were probably related to a chronic intoxication owing to the ingestion of toxic plants. In addition, tissue samples from this animal were used for bacteriology, yielding negative results. Animals 2 and 3, which had attained inconclusive results in the P22 ELISA in a previous testing event (February 2017), did not have any clinical signs compatible with TB infection and were not, therefore, euthanized. Nevertheless, the oldest animal in the herd was weak and had a poor body condition, and was euthanized (Animal 5). However, no compatible TB lesions were found at necropsy and the bacteriological culture from tissues was negative.

#### October 2018

One animal died of natural causes (Animal 6) and was subjected to necropsy. This animal did not have TB-compatible lesions at necropsy, and the bacteriological culture was negative.

#### March 2019

One year later, all the animals present in the herd (*n* = 20) were negative to all the TB diagnostic techniques used (SIT test, CIT test, DPP and P22 ELISA).

## Discussion and conclusions

Few studies concerning members of the MTBC causing TB outbreaks in OWC have been published, and *M. bovis* was identified in most of them [2–6, 9, 10, 22], excluding one case of an *M. caprae* infection in a zoo [11]. To the best of the authors’ knowledge, the present study is the first to describe an *M. caprae* infection in a farmed OWC, thus providing the opportunity to increase the knowledge concerning TB in camels.

In the present study, only one animal was positive to the CIT test and was subsequently confirmed as *M. caprae*-infected by post-mortem analysis and bacteriological culture. Another animal that was a positive reactor to the SIT test (but negative to the CIT test) was also preventively euthanized. However, non-compatible TB-lesions were observed and no species of the MTBC were eventually isolated. According to previous studies, the CIT test is more specific and usually preferred over the SIT test in camelids, since non-specific reactions have been reported when using the SIT test, probably owing to infection by atypical mycobacteria [8]. Nevertheless, the use of the CIT test may entail a lower probability of TB detection owing to its lower sensitivity [10], and its use should, therefore, be avoided when the presence of TB is suspected. With regard to serology, the TB-infected animal was negative to DPP but positive to the P22 ELISA, thus suggesting a more limited sensitivity of DPP in comparison to the experimental ELISA [4, 15]. The differences between these two serological tests may be owing to the antibody detection system, since DPP uses protein A/G linked to horseradish peroxidase (HRP), which has less affinity to antibodies than an anti-llama HRP secondary antibody employed in the P22 ELISA. Animals 2 and 3, whose ELISA results were inconclusive, were negative in the subsequent herd testing event, suggesting that the cut-off should be re-adjusted or the occurrence of cross reactivity with MAC [8]. The reactivity against avian PPD of another two camels in the herd (data not shown) may indicate that the animals were sensitised against proteins from *M. avium,* some of which could be included in the P22 protein complex [18]. However, these two camels were not euthanized, since there was no clear clinical evidence of TB infection in them, although one of them had a lesion caused by *C. pseudotuberculosis*. This bacterium has been related to interferences in the diagnosis of TB in other species, and may also explain certain positive reactions in the herd [23]. This limited number of animals subjected to post-mortem analysis signifies that an accurate evaluation of the performance of the various diagnostic tests was not possible, although the results might indicate that a parallel interpretation of CIT and ELISA would be valuable for TB control in OWC, as has been demonstrated in the case of SAC [19, 24].

It should be noted that the herd was subjected to a close surveillance when Animal 1 was confirmed as TB-infected, and no animals were found to be positive to any of the diagnostic tests in subsequent testing events, and neither were the other three animals that were subjected to post-mortem analysis (two euthanized and one natural death) confirmed as TB infected. It is, therefore, possible to conclude that the *M. caprae* infected camel (Animal 1) was the only case in the herd, and that no transmission to other animals in the herd occurred. Several hypotheses could explain this absence of transmission to other animals in the herd (low or absent bacterial shedding by the respiratory route, presence of the primary complex in the abdominal organs, absence of close contact, etc., although the presence of a lower severity of lesions in the lungs with a low number of bacteria in comparison to the other organs would be determinant. The clinical signs are, according to other authors [7], vague or non-existent. In our case, Animal 1 underwent behavioural changes and its body condition deteriorated owing to its progressive loss of appetite and weight loss, and was, in the final phase, intolerant to exercise. The type and distribution of the TB lesions observed in the infected animal studied herein were similar to those described in previous studies on *M. bovis* infected camels [9, 10, 13, 22], suggesting that *M. caprae* caused a clinically and histologically indistinguishable infection. Nevertheless, the aforementioned authors noted three different gross patterns related to the distribution and incidence of TB lesions in dromedary camels: pulmonary, abdominal and disseminated forms. Our animal had the less common form characterised by extended granulomatous nodes in both the abdominal and thorax cavities. Little data concerning *M. caprae* infection in OWC is available, and further studies are necessary to demonstrate whether the infection by different members of the *M. tuberculosis* complex may affect the evolution and diagnosis of the infection in OWC, as has been suggested in the case of other animal species [25].

According to the epidemiological data provided by the owner, the consumption of raw goat’s milk by the camel was the most likely cause of the TB infection. The whole herd, 22 animals, was imported from OTF regions to Spain, and only two animals were born on the farm. The infected animal (Animal 1), one of those, was fed with raw goat’s milk from a goat herd located in a region of high caprine TB prevalence, and it is feasible that mycobacteria was present in the milk [26]. In fact, *M. caprae* SB0157 is the most predominant spoligotype in goats in Andalusia, and has been isolated from other species such as cattle, sheep, pigs and wild boar [27, 28]. Unfortunately, the goat herd was removed prior to this study and it was not, therefore, possible to confirm this hypothesis, which was supported by the presence of TB lesions in the abdominal organs, where primary TB complex was localised (digestive primary complex), suggesting the oral route of infection. The infection was later generalised to different organs, including the lungs. This might be why the lesions were more advanced in the spleen and liver than in the lungs. The pulmonary lesions were, therefore, small and well demarcated round lesions with abundant caseum material, probably at an early stage, and had a low bacillary load. These findings suggest a limited or absent capacity to transmit the infection by the respiratory route.

In conclusion, the present study provides the first description of disseminated TB with a digestive primary complex caused by *M. caprae* in a farmed OWC. Although the origin of the infection could not be accurately determined, the consumption of raw goat’s milk was considered the most feasible hypothesis. Moreover, this study shed light on the performance of TB diagnostic tests in OWC, suggesting that a combination of the intradermal tests with P22 ELISA is a valuable option, although this should be confirmed by using a higher number of samples.

## Supplementary Information

**Additional file 1.**

## Data Availability

The datasets that support the findings of this article are included within the article.

## Abbreviations

CIT: Comparative intradermal tuberculin
DPP: Dual Path Platform
DVR: Direct Variable Repeat
MAC: *Mycobacterium avium* Complex
MTBC: *Mycobacterium tuberculosis* Complex
OD: Optical density
OTF: Official Tuberculosis Free
OWC: Old World Camelids
PPD: Purified Protein Derivative
SAC: South America Camelids
SIT: Single intradermal tuberculin
TB: Tuberculosis
